# Nasopharyngeal Swab for COVID-19 Test Necessitating Mechanical Ventilation and Tracheostomy

**DOI:** 10.7759/cureus.13908

**Published:** 2021-03-15

**Authors:** Resha Khanal, Sharad Oli, Halimat Lawal, Binita Bhandari, Saketram Komanduri

**Affiliations:** 1 Internal Medicine, University of Pittsburgh Medical Center Pinnacle, Harrisburg, USA; 2 Internal Medicine, Maimonides Medical Center, Brooklyn, USA

**Keywords:** nasopharyngeal swabbing, sars- cov-2, epistaxis, mechanical ventilation, tracheostomy

## Abstract

We present the first-ever reported case of massive epistaxis following nasopharyngeal (NP) swabbing requiring intubation and tracheostomy. A 67-year-old male with a mechanical aortic valve on warfarin presented from a nursing home to the emergency department with hypoxia. NP swab for coronavirus disease 2019 (COVID-19) was obtained, immediately followed by significant epistaxis. Patient desaturated to low 80s requiring intubation for airway protection and hypoxemic respiratory failure. Anterior nasal packing was performed. The COVID-19 test resulted negative. Extubation was unsuccessful on days four and nine. The patient subsequently underwent tracheostomy and percutaneous endoscopic gastrostomy (PEG) tube placement. The patient was transferred to sub-acute rehabilitation with a tracheostomy tube on minimal ventilator support.

The World Health Organization (WHO) has recommended obtaining an NP swab in COVID-19 suspects to test for severe acute respiratory syndrome coronavirus 2 (SARS-CoV-2) using reverse transcriptase polymerase chain reaction (PCR).A study found that NP swabbing was associated with epistaxis in approximately 5-10% of the cases. Nursing home populations are at higher risk for COVID-19 and also reported to have increased use of oral anticoagulation for chronic atrial fibrillation with other co-morbidities (high CHADVASc score) which may increase bleeding risk with NP swabbing. Less invasive methods such as salivary and mid-turbinate sampling, nasal swab or saliva can be a better alternative sample for detecting SARS-CoV-2 as recommended by the Centers for Disease Control and Prevention (CDC) and suggested by FDA. Positive PCR testing beyond nine days of illness is likely due to persistent dead virus particles and thus repeat testing is not suggested. Obtaining a history of bleeding diathesis, use of oral anticoagulants and consideration of NP anatomy is advised before swabbing.

This case report raises the concern against inadvertent NP swabbing in cases with a low pretest probability of COVID-19 infection with higher bleeding risk.

## Introduction

The severe acute respiratory syndrome coronavirus 2 (SARS-CoV-2) test consists of obtaining a nasopharyngeal swab specimen for polymerase chain reaction (PCR) testing. Here we present, to the best of our knowledge, the first-ever reported case of massive epistaxis requiring intubation and tracheostomy following nasopharyngeal swab collection for coronavirus disease (COVID-19) testing.

## Case presentation

A 67-year-old male with a history of chronic obstructive pulmonary disease (COPD), mechanical aortic valve on warfarin, coronary artery disease, and diastolic heart failure was brought to the emergency department (ED) from a nursing home for hypoxia. ED vitals were significant for oxygen saturation of 87% on room air. Physical examination was notable for 2+ bilateral pitting pedal edema. Significant laboratory values included an international normalized ratio (INR) of 2.6 and platelet count of 113 K/microL. Beta natriuretic peptide was 281 pg/ml. Chest X-ray at presentation showed hypoinflation with bilateral diffuse interstitial opacities suggestive of volume overload. The patient received 40 mg of intravenous furosemide followed by improvement of the respiratory symptoms. A nasopharyngeal swab was obtained for COVID-19 testing, which was immediately followed by significant epistaxis. Hypoxia worsened and he was placed on 15 liters of oxygen via a non-rebreather mask. He continued to desaturate to the low 80s. The patient was intubated for airway protection and worsening hypoxia and was transferred to the intensive care unit. Warfarin was held. Ear Nose and Throat (ENT) confirmed the bleeding from the superior and middle turbinates and anterior nasal packing was performed. The COVID-19 test was reported to be negative twice during the hospital stay. The patient was extubated on day four, however, the patient went into atrial fibrillation with a rapid ventricular rate leading to hypoxia. Arterial blood gas (ABG) showed a pH of 7.3 and partial pressure of carbon dioxide (pCO2) of 68 mmHg. The patient was re-intubated after he was found to be unresponsive and desaturating to 79% on 40L 100% Vapotherm. On day nine of the hospital stay, the patient was extubated again followed by re-intubation within few hoursfor sustained ventricular tachycardia leading to hypoxia. The patient underwent tracheostomy and percutaneous endoscopic gastrostomy tube placement on day 12. The patient’s hospital course was complicated by pneumonia which was treated with broad-spectrum antibiotics. The patient was then transferred to the medical floor. On day 25, he was found to be pulseless and cyanotic with agonal breathing. Pulseless electrical activity (PEA) was identified in the rhythm strip and advanced cardiac life support (ACLS) protocol was promptly initiated followed by achievement of spontaneous circulation. After 72 days of hospital stay, the patient’s acute issues resolved and he was transferred to subacute rehabilitation with a tracheostomy tube on minimal ventilator support.

## Discussion

The importance of accurate screening of COVID-19 suspects in this pandemic is well established. The WHO has recommended obtaining a nasopharyngeal swab to test for SARS-CoV-2 using reverse transcriptase PCR (RT-PCR)-based assays [1]. The swab is roughly 15 cm long and the swab’s head is coated with synthetic filaments. It is inserted deep inside the nostril where it hits the back of the nasopharyngeal cavity. In a study done by Gupta et al., it was found that nasopharyngeal swab collection was associated with epistaxis in approximately 5-10% of the cases. Other minor side effects included headache, earache, and rhinorrhea lasting for a few hours [2]. The swab tip needs to be smooth enough for patient comfort and to avoid epistaxis. Patients on anticoagulation are likely at higher risk for epistaxis like in our case and should have prompt consideration of whether withholding anticoagulation or reversing the agent would be beneficial. Treatment of epistaxis consists of securing the airways and maintaining hemodynamic stability as the initial approach. This is to be followed by conservative measures such as prolonged nasal pinching, chemical or electrical cauterization, and/or nasal packing if the above measures fail. To the best of our knowledge, this is the first reported case of epistaxis leading to endotracheal intubation followed by tracheostomy after nasopharyngeal swab testing.

Nursing home populations are considered to be higher risk groups for COVID-19 and hence undergo higher numbers of COVID-19 nasopharyngeal swab testing as in our patient. It is reported that the number of nursing home residents on oral anticoagulation due to chronic atrial fibrillation has increased to 48% in 2016 as compared to 42% in 2011 [3]. Hence, this patient population may have an increased risk of epistaxis. Less invasive methods of testing for COVID-19 such as salivary and mid-turbinate sampling approaches are upcoming alternatives with fewer complications [2]. As reported by Pere et al., nasal swab specimens showed equivalent accuracy in detection of SARS-CoV-2, thus can be used as an alternative to nasopharyngeal swab [4]. A study by Wyllie et al. concluded that saliva can be a better alternative sample for detecting SARS-CoV-2 given its higher sensitivity and detection consistency as compared to nasopharyngeal swabs, as well as being a less invasive procedure [5]. The Centers for Disease Control and Prevention (CDC) on October 2020 recommended alternate specimens like nasopharyngeal wash/aspirate, nasal wash/aspirate, oropharyngeal (OP) swab, mid-turbinate (MT) swab, anterior nares (AN) nasal swab, and saliva for COVID-19 testing. The FDA also suggested the use of OP, MT, and AN as alternate specimens for COVID-19 testing [6]. Nasopharyngeal anatomical consideration is to be kept in mind to prevent complications including epistaxis and patient discomfort during procuring nasopharyngeal samples [7]. It is advised to obtain a patient's history of bleeding diathesis or the use of oral anticoagulants. 

A recently published systematic review and meta-analysis including more than 5,000 study individuals showed that live virus was not detected after nine days of illness. Therefore, positive PCR testing beyond that time frame is likely due to persistent dead virus particles that are non-infectious and thus the study suggests against repeat testing to verify the COVID-19 status [8].

## Conclusions

This case report is written to emphasize the possible worst outcome of obtaining nasopharyngeal swabs leading to multiple in-hospital complications. In high-risk cases such as ours, swab collection with extra precautions, the involvement of experts, or alternative options like obtaining nasal swab or nasopharyngeal aspirate could be better options. This case report has also raised the question against inadvertent and routine nasopharyngeal swabs in cases with a low pretest probability of COVID-19 who have a high bleeding risk.
